# Report on Practical Obstetrics for the Year 1858

**Published:** 1859-05

**Authors:** J. K. Mason

**Affiliations:** Fellow of the Philadelphia College of Physicians, Lecturer on Midwifery, etc.


### REPORT ON PRACTICAL OBSTETRICS FOR THE YEAR 1858.

By J. K. MASON, M.D., Fellow of the Philadelphia College of Physicians, Lecturer on Midwifery, etc.

THE purpose of this report is to give a summary of all the original papers on the subject, published during the year 1858, in the United States. This has been done by the reporter, so far as circumstances would permit.

Some articles treating of purely theoretical points, more interesting to the disputant than to the practical obstetrician, have been omitted, not that the author thinks such discussions useless or unnecessary, but because his object has been to make this report as practical as possible. Some interesting papers have doubtless been passed over, and others mentioned only in a cursory manner, whose merits entitle them to a wider space than can always be afforded in such a work as this, in which condensation is of so much importance.

*Case of Fallopian Pregnancy resulting in Rupture of the Cyst, and terminating in Death.* Reported by ROBERT P. HARRIS, M.D., Philadelphia. Read before the Philadelphia Pathological Society, November 25th, 1857. Contained in the *American Journal of Medical Sciences*, January, 1858.—Mrs. S., the subject of this report, is stated to have been a lady of medium height, somewhat full habit, twenty-seven years of age, and the mother of an infant a year old. On the morning of the 24th of October, 1857, was apparently in perfect health, and between nine and ten o'clock, shortly after breakfast, was suddenly seized with excruciating pain in the lower part of the abdomen, followed immediately by a feeling of faintness. Her pulse became very rapid and feeble, extremities cold, and surface of the body very pale, as though exhaustion were being produced by internal hemorrhage. Every sort of stimulation was used, but to no purpose. The pain, which at first was very severe, soon diminished, and finally ceased. She gradually became more and more feeble, so that in a few hours from the commencement of the attack, all hope of recovery was abandoned. She remained in a moribund condition, with little apparent change, until, from a gradual loss of vital power, she ceased to breathe about seven o'clock on the following morning, twenty-one hours after the commencement of the attack.

The post-mortem examination of this case, which was with some difficulty obtained, is interesting; we shall, therefore, give Dr. Harris's description of it:—

"Upon uncovering the body, the abdomen was found much distended and tympanitic over its whole extent; and incising the skin and soft parts down to the linea alba a thickness of adipose deposit, varying from an inch to an inch and a half, proved the full state of health of the patient at the onset of her disease. Further dissection showed that the intestines, both large and small, were very much inflated, and that the omentum was wrinkled and drawn up to the right upper portion of the umbilical region.

"Extending the vertical incision down to the pubis, we were arrested in our examination by the escape of a small quantity of serous fluid, and upon drawing the intestines upward from the brim of the pelvis, we found that the cavity of the pelvis, as well as the lower and back part of the abdominal cavity, were filled with blood in a partly fluid, partly coagulated state. After this was removed by means of a sponge, to the amount, perhaps, of half a gallon, a small abnormal growth in the course of the right Fallopian tube arrested our attention, and caused us to dissect out and carefully remove the uterus with its appendages. The uterus proved to be scarcely, if at all, enlarged beyond the size we usually find it in healthy women who have borne children. Its cavity contained a little mucus slightly tinged with blood, but no trace of a membrana decidua; the ovaries were healthy, of normal size, but very pale in color, from their anæmic state; the left Fallopian tube presented nothing unusual; but in the right one, immediately above the ovary, was a tumor of an oval form, slightly flattened antero-posteriorly, about an inch and a quarter long, an inch in its vertical diameter, and of a reddish-blue color. Upon close examination, a small opening of a little more than a line in diameter was detected at its upper surface, perforating the edge of the broad ligament. This hole communicated with the peripheral portion of the body, but not with its interior cavity. An incision made through the cortical portion, so as to divide the body to its centre, revealed a body containing a small diaphanous sac, within which was a human embryo of a pure white color and half an inch in length, looking a good deal like a common maggot."

The chief peculiarity of this case was the very early age in the development of the ovum at which the rupture took place, but the accident is plausibly accounted for by the reporter, who states that the female was menstruating at the time. In his own words: "The increased flow of blood to the uterine organs probably contributed to produce the rupture of the cyst, which appeared to have given way very much after the manner of an aneurismal sac."

Dr. Harris justly remarks that the tubal or Fallopian is by far the most frequent of all the extra uterine pregnancies; he then discusses the cause of Fallopian pregnancy, and remarks on the great difficulty of diagnosis in some of these cases. But we have no space for further extracts.

Dr. Harris thinks that little can be said that is favorable to the operation of gastrotomy in extra uterine pregnancy; he is borne out in this opinion by Cazeaux and Moreau, who are, as he remarks, strongly opposed to the operation, regarding it merely as a last and desperate effort of professional duty to save a patient whom no other means can reach.

In the same number of this journal, page 44, is

*A Case of Extra Uterine Pregnancy.* By CHRISTOPHER JOHNSTON, M.D., Surgeon to the Union Protestant Infirmary, Baltimore.—The fœtus was in this case extracted per anum, four years and six weeks after the completion of the natural term.

The amazing efforts of nature to work out her own cure are here well exemplified. On the 18th of March, 1858, Mary Hamilton, aged thirty-nine, was admitted into the Union Protestant Infirmary of Baltimore. Had supposed herself laboring under dysentery for ten weeks previously, and was at the moment of her admission extremely emaciated, and much annoyed by abdominal pains, and frequent small muco-sanguineous intestinal discharges, always offensive, and at times mingled with dark semi-fluid fæces. A week after her admission, the patient was placed under the care of Dr. Johnston. The treatment she had been under (not stated what) was continued and modified in various ways, without success; thus particular attention was not paid to the local difficulty until the sixth of April, when the invalid complained of a something which pressed painfully upon her lower bowel. The doctor then made digital examination of both vagina and rectum. He discovered the os tincæ to be very low in the pelvis, but not otherwise displaced; the uterus seemed to extend suddenly from the neck into a a considerable tumor, which could be felt through the abdominal walls in the right iliac region. The following is Dr. Johnston's account of the result of his exploration:—

"Within the anal sphincter the same tumor could be perceived through the thickened wall of the gut; but at the depth of a finger's length, a culminating point was found, and upon this an opening was made an inch and a half wide by an inch in length, in which was impacted a mass of long plates, with here and there a thin sharp edge. I at once recognized, I believed, fœtal cranial bones; and upon inquiring of the patient if she had not at some time supposed herself pregnant, she replied in the affirmative, but added that that was four or five years since; and as no child was born, the enlargement was thought to be a tumor, and the idea of pregnancy discarded. In fact, from the time of expected confinement she had felt 'a lump' in her side, (which I also discovered,) but she suspected no connection between the tumor and her dysenteric attack; she thought, and her friends thought, that she had dysentery," etc. etc.

The woman's account of herself and her case is extremely clear and precise, so much so, that the doctor says that it strongly corroborated his diagnosis, and he at once proceeded to remove, by artificial means, the mass of bones and flesh which the tumor contained.

The sac contracted notably after the extraction of the contents. About six ounces of blood and a greater quantity of putrid ooze flowed out of the gut; but there was no after-hemorrhage.

"The after-treatment consisted in a light nourishing diet, a nightly anodyne, the employment of the catheter, and an ablution of the rectum at bedtime with warm water. The patient is rapidly improving without an untoward symptom; and the discharge, which at first was insufferably fetid, has become almost purulent and nearly inoffensive."

Thus this poor woman became pregnant in May, 1853, she quickened at the usual time, and distinctly felt the motions of the child; on the 3d of January, 1854, she was seized with what seemed to be the pains of labor, and the attending physician believed that she would soon be delivered. The movements of the child were felt up to the sixth, when, as the patient herself very graphically expresses it, "I experienced a sensation as if the child stretched itself out, and thenceforth all motion ceased." After-pains continued for three weeks, in spite of treatment, at the end of which time she left her bed and returned to her duties, but suffered much, as may easily be supposed, from ill health, being constantly racked with pains in the abdomen and back until the night after Christmas, 1857, she became very sick with what she thought to be dysentery, which persisted with great obstinacy until worn completely out, emaciated to a skeleton. She entered the Infirmary on the 18th of March, 1858.

Dr. Johnston is satisfied that this was a case of true abdominal pregnancy, and all the facts seem to confirm his opinion, for by the mother's account, the motions of the child were distinctly felt up to the eighth month, an impossibility, we should say, had the pregnancy been interstitial, tubal, or ovarian. His remarks are as follows: "I have now satisfied myself, by digital examination, that the fœtal sac is distinct from the uterus, although adherent to that organ—that is to say, the pregnancy was not parietal."

The patient is convalescing rapidly.

*Case of Extra Uterine Pregnancy, Complicated with General Anasarca and Ossification of the Uterus.* By R. W. HARDEE, M.D., Savannah, Georgia.—This young physician here describes a very interesting case with great truth and simplicity, and with a modesty which in the present age is as surprising as it is refreshing. Dr. Hardee was called upon some months ago, to visit a negro woman belonging to a neighbor. His first impression, from her general appearance, was that he had a very obstinate case of anasarca to deal with, but on examining the abdomen, he felt a large tumor resting upon the left side, directly on a line with the umbilicus. Upon a per-vaginal examination he found the uterus presented to the touch the sensation of a hard bony mass; no os tincæ to be found. She was in great pain, and suffering from general dropsy, particularly of the abdomen.

The diagnosis was obscure; all that the doctor could learn from the patient was, that the tumor had been growing in her side for fifteen years; did not remember having ever met with any injury or accident. Under these circumstances, Dr. Hardee consulted with his friend Dr. Steele, and, after mature deliberation, they decided it to be a case of extra uterine pregnancy.

"Prognosis very unfavorable." She was treated for the dropsy, with mercurials, purgatives, diaphoretics, and diuretics; purgatives seemed to give her great temporary relief. At length she increased so enormously, that tapping was absolutely necessary, which operation was repeated once a week for about a month. The author does not state the precise time of her death, but it must have been very shortly after this. Autopsy six hours after death. "On laying open the abdomen, the uterus and a fœtal head, larger than at term, were brought to our view. The head resting just below the navel, and on the left side of the body; it was firmly attached to the uterus and intestines. After removing the head, we saw a decayed mass, but could not discover what it was. All the bones of the fœtal head were present, with the exception of the superior and inferior maxillaries. On opening the head, we could see the brain and all its divisions. The uterus was about ten inches long, about four inches wide, and four thick, forming one hard bony mass, weighing six or eight pounds."—(*Oglethorpe Medical and Surgical Journal*, August, 1858, p. 138.)

*Case of Tubal Pregnancy, with Rupture and Death.* By G. G. STEELE, M.D., Huntsville, Alabama.—The following brief history of a very interesting case was communicated to Professor Pope, by the author, in a letter not designed for publication: "I sent you yesterday by express, a pathological specimen, which, I hope, will be an acceptable addition to your museum. It is a case of tubal fœtation, taken from a servant-woman of about twenty-six or twenty-eight years of age, and of unusual good health, (second pregnancy;) first child eight or ten years old; married a second time about two months since. Two weeks prior to her death, she missed her courses for the first time, and suffered no pain up to the night of the seventeenth, when she was taken with severe pain of the abdomen, which was supposed to be bilious colic, and for such treated by the family. The next day, about ten or eleven o'clock, I saw her, when she said she was better; I found, however, her pulse small, quick, and feeble, and still complaining of a pain of a spasmodic character over the whole abdomen; but as she said, not near so bad as the night before, I was at a loss to make out what it was, or to form a diagnosis. I ordered some brandy-toddy, warm applications to the abdomen, and a warm enema. She died suddenly about an hour after my seeing her. I was so shocked by the suddenness of her death, that I could form no opinion as to its cause; and it was not until that night after going to bed, and carefully reviewing all the symptoms, that I could settle upon an opinion; I am glad to say that opinion was correct, and I expressed my belief to the other physicians before going to make the examination, that it was tubal pregnancy, with rupture. This opinion, or diagnosis, was formed by exclusion; by seeing what it was *not*, I must candidly acknowledge that it was but a good guess. On opening the cavity of the abdomen, I found effused about a gallon and a half of blood; that in the pelvic cavity was coagulated, in removing which I found an embryo of about six weeks, lying near the rupture in the tube, which was about midway. The embryo, with the uterus and appendages, I inclosed, but fear it will be destroyed before reaching you. I am fully persuaded that this accident can happen oftener than is supposed. Unless hemorrhage is severe, recovery could easily take place without producing any subsequent alarming symptoms. In the present case, death was the result of hemorrhage. I can see no reason why fatal, or even severe hemorrhage, should always follow. It is only after an autopsy that the diagnosis is made out, and as this does not often occur, the accident is considered one of rare occurrence." —(*St. Louis Medical and Surgical Journal*, November, 1858, p. 568.)

The author's suggestion that this accident may occur more frequently than is generally supposed, and that the woman may often recover where the hemorrhage is slight, without any violent symptoms, we think very rational.

*Report of a Case of Extraction of the Bones of a Fœtus from the Peritoneal Cavity, after having been there over Four Years.* By D. W. YOUNG, M.D., Aurora, Illinois.—This case is extremely interesting, from the fact that the lady became pregnant twice, carried her children to full term, and was delivered of two fine healthy infants, carrying at the same time, a child at full term encysted in the cavity of the peritoneum. On the 17th of August, 1855, Dr. Young was called to attend a robust German lady, residing near the city. She was delivered, without difficulty, of a fine, well-formed male child. After all was over, the lady directed his attention to a tumor in her right side, situated in the iliac region, which she informed him had been there for something over four years, and gave the following history of its commencement: "While still residing in Germany, she became, as she supposed, pregnant for the first time; that she enjoyed pretty good health during the first six months of gestation, but during the latter three months, she suffered a good deal of pain in her right side. At the expected time for her confinement, pains came on, and she and her friends supposed that labor had really commenced, and accordingly called their midwife, who came, and after repeated examinations, expressed her fears 'that the child did not lie right,' as she could not reach it, and as it did not advance properly notwithstanding the severe pains. Her pains continued and increased, until, she says, something burst, and discharged a large quantity of water and bloody matter, together with clots of coagulated blood, after which the pains ceased; the tumor diminished in size and moved higher up, and further to the right side. The final conclusion with them was, that she had only suffered from obstructed menstruation which had now been relieved. The lochial discharge continued for two or three weeks, during which time the tumor also diminished somewhat in size. Finally the lochial discharge ceased, leaving the present tumor, which to all appearance has remained dormant up to this time.

"She afterwards consulted a surgeon about the tumor, who said he thought it was a disease of the ovary, which as long as it did not trouble her very much, she had better leave untouched. She soon again became pregnant, and in due time was delivered of a healthy child. Parturition was natural and easy, and she soon regained her usual degree of health, having experienced nothing different from other parturient women, except an entire absence of the lacteal secretion. 'The tumor apparently remaining in the same dormant condition.'" Some two years subsequently, she again became pregnant, when she came under the care of Dr. Young, who delivered her, August, 1855, of a fine healthy child. "The labor uncomplicated and easy." Dr. Young thus describes the termination of this case:—

"I heard nothing more of Mrs. Yomgels, until the 1st of May, 1856, when her husband called upon me at my office, to consult me in relation to his wife, who, he said, was very poorly. He informed me that some time after her confinement, she was attacked with chills and fever, and that as his friend, A. W. Heise, M.D., a very eminent German physician and surgeon, from Addison, Du Page County, Illinois, was in the neighborhood, he called him to prescribe for his wife. Notwithstanding the medicine, the chills and fever continued; the tumor became painful, inflamed, and finally opened, and discharged several small bones, one of which he exhibited, and I pronounced it to be the fibula of the right side. He said that he had that day written to Dr. Heise, requesting him to visit his wife immediately, and desired that I would see her with him when he came. I promised, and he returned home. On the 5th of May, 1856, I was summoned to meet Dr. Heise in consultation, who was then waiting at her house. At my request, Dr. P. A. Allaire, of this city, was also called to examine the case in consultation. The patient was much emaciated, and seemed suffering from hectic fever and severe night-sweats, together with extreme nervous prostration. The tumor near the umbilicus was ulcerated, and discharging a most terribly offensive matter. After due consultation, and, at the earnest solicitation of the patient, an operation was decided upon, notwithstanding her extreme emaciation and feebleness. Accordingly I put the patient thoroughly under the influence of chloroform, when the operator, Dr. Heise, proceeded, with a groved director and bistoury, to make an incision of some inch or two toward the right side, and with a long pair of forceps commenced to extract the bones. The bones of the head and pelvis were too large to pass through the small orifice, which seemed as large as we dare make for fear of cutting beyond the adhesions of the sack and peritoneum, consequently they were broken up with a strong pair of scissors. Drs. Heise and Allaire took turns in operating, and were full two hours in removing all the bones and cleansing the sac. The chloroform acted so admirably, that when the operation terminated, and she was permitted to come from under its influence, she expressed herself as feeling decidedly better.

"The sac was firm, dense, and so tough, as almost to resist the edge of the scalpel, and was contracted closely around the bones. After the operation, the dressing consisted of a simple broad bandage around the abdomen, with directions that she be kept on her side, so as to facilitate the discharge from the wound." This case progressed favorably for four or five days, when the author discovered that nearly all the fæces passed by the wound, and through the orifice of the sac; this formidable complication was combated by compresses and bandages, and soon disappeared; she rapidly improved under the use of tonics and nutritious diet, and Dr. Young had the satisfaction of discharging her perfectly cured at the end of four months.

Our own impression is, that this was a case of rupture of the womb, and not of extra uterine pregnancy. The patient feeling something give way during her pains and the escape of blood and clots from the vagina at the same time, seem to favor this opinion; how the woman escaped death from violent peritonitis, is very remarkable.—(*From the Chicago Medical Journal*, August, 1858, p. 383.)

*Rupture of the Uterus during Labor.* By M. F. BROWN, M.D., Hannibal, MISSOURI.—This was the case of a young negress, who, during her first pregnancy, complained to the doctor of a tumor, which appeared externally at the vulva. Upon making an examination, he found it to be the neck of the womb "enormously elongated."

She was kept in a recumbent position, until labor came on; the pains were severe, and the vertex of the child appeared externally, covered by the neck of the womb, unyielding, but much attenuated. "At length, during a sharp pain, the neck of the womb gave way in a straight longitudinal laceration on the left side, extending up to the body of the organ, but not high enough to penetrate the peritoneal cavity." After the placenta came away, the torn edges of the neck of the womb could still be drawn down beyond the external genitals. "No unusual hemorrhage followed." The patient appears to have been treated with great prudence and faithfulness, making a rapid recovery, without a single unfavorable symptom.

The doctor writes: "The edges of the laceration were united by granulation, and the neck of the womb appears to be in the natural position. The woman, at this time, thirty-two days after the accident, is going about attending to her ordinary duties, and is evidently in the enjoyment of most perfect health."—(*St. Louis Medical and Surgical Journal*, January, 1858, p. 39.)

*Report of a Case of Monstrosity.* By J. E. G. TERRILL, M.D., Greenville, Georgia.—This woman, a negress, was threatened with abortion, (at what period of time, before full term, the reporter does not state;) by treatment the accident was prevented, and she afterwards was able to attend to her duties on the farm. May the fifth, she was delivered without difficulty of a singularly formed child, the description of which we quote from Dr. Terrill's report: "The head is regularly formed, with quite a sufficiency of hair; so are the upper extremities and chest. The abdominal walls are wanting, with the exception of a small space above the pubes; the liver and intestines are exposed fully to view, hanging loosely, and retained in situ by the reflections of the peritoneum; the lower extremities are reversed: they fold upon the back instead of the abdomen. The anterior portions are in this case the posterior portions of the legs; the os calcis is in front, the toes pointing backward; the great toes are upon the outside of the feet; the feet are flat, being made so doubtless by their position upon the occiput while in utero. No signs of genitals, either male or female; the parts are perfectly smooth. The anus is above the os pubis, just below the margin that surrounds the viscera of the abdomen. The bladder is not visible, and the spine is considerably distorted. The child's weight is about seven pounds; it was still-born. I have not made any dissection, as I wish to preserve the specimen and present it to the Museum of the Atlanta Medical College."—(*Atlanta Medical and Surgical Journal*, July, 1858, p. 651.)

*Difficult Labor; Monster Craniotomy without Instruments.* By N. L. NORTH, M.D., Brooklyn, New York, Consulting-Physician to the Williamsburg Dispensary, etc.—This woman, Mary McVey, had been in labor many hours, was consumptive, and in a sinking condition. The forceps were applied and easily locked, but the head was so soft, that upon traction they continually slipped. Dr. North did not think it advisable to wait for the arrival of craniotomy instruments, but by pressing the scalp against the edge of one of the parietal bones, contrived to make it cut through the integuments and muscle, when an enormous quantity of sanguino-serous fluid escaped with a gush, the head collapsed, and the child was born without further trouble. The mother recovered without an untoward symptom. "The child was a rare sight. It had a double harelip. With the exception of two slight prominences just above the angles of the mouth, the whole middle portion of the upper jaw, to the malar bones each way, and the whole of the roof of the mouth and the septum nasi, were wanting; the nose itself looked like the upper lip, except a slight cartilaginous protuberance in the centre, seeming to be an attempt at the formation of the septum nasi. The size of the head, as near as we could calculate, (it being emptied of its contents,) was as follows: occipito-mental, ten inches; occipito-frontal, eight and a half inches; biparietal, six and a half inches; cervico-bregmatic, five inches."—(*Buffalo Medical Journal and Monthly Review*, August, 1858, p. 149.)

*Two Fœtuses United Face to Face—from the Umbilicus to the Upper Part of the Sternum.*—This case is reported by J. B. S. JACKSON, M.D., Boston, who obtained the specimen for dissection, and an account of the labor from Dr. D. J. Perly, Old Town, a few miles from Bangor. We shall not attempt to quote Dr. Perly's description of the labor, which was difficult and dangerous, and appears to have been extremely well managed. There was but one placenta and one cord, until within about two inches of the fœtal abdomen, where it bifurcated to supply each of the children. The cord pulsated strongly, and both children were alive at birth, but animation soon ceased. Dr. Jackson gives a description of his dissection, with the size and relative position of the different organs. It is highly interesting, but we regret that our limited space prevents our making extracts.—(*Boston Medical and Surgical Journal*, June, 1858, p. 274.)

*Account of a Monster of the Genus Peracephalus.* Read before the Philadelphia Biological Society, March 1st, 1858, by WALTER ATLEE, M.D.—"This monster was the product of a double gestation, the mother being delivered at seven months. The child born first lived fifty-five hours; it was a female, and perfect, with the exception of one club-foot: the other monster came away about twenty minutes afterwards. One placenta was common to both. The mother was a healthy woman, who had previously borne three children in as many confinements. In the first two pregnancies the children were females, and born at seven months; in the third the child was male, and born at eight months and a half. The woman had received, as far as is known, no fall or fright in the course of her pregnancy. The monster has no head or upper extremities. The trunk and lower extremities are of a size corresponding to those of a well-formed fœtus of seven months. A want of perfect symmetry, however, is very manifest in the two halves of the trunk, which is remarkable for a number of elevations and depressions between them. These elevations are due to the accumulation of a great quantity of cellular tissue. This sort of lumpy condition exists, likewise, in the lower limbs, which present, in addition, several other imperfections. The feet are turned inward, and they possess but four toes, two of which, the smallest, on one foot are not separated. The external organs of generation are those of the female; the anus exists upon the front of the trunk; between the umbilicus and its upper extremity is something like the appearance of a small empty bag or bladder, attached by a rather narrow pedicle. Both above and below this bag is a small tuft of hair. Toward the sides of the thorax, in the places usually occupied by the nipples, are small round holes.

"For the sake of preserving the original appearance of this monster, which was deposited in the collection of the Biological Society of this city, the examination made of its internal structure was very limited; it was confined, in fact, to an examination of the spinal column. The sacrum was found to have its concavity looking backward; the lumbar vertebræ are five in number, and the dorsal twelve; at the commencement of the cervical region the spine is bent forward and downward, and it leads by a chain of small, rudimentary bones, imperfectly formed, but evident rudiments of vertebræ and a cranium, to the empty bag, mentioned as existing in the front of the thorax. The existence of a few hairs on this place, rudiments of the hairy scalp, had already foretold, what a a slight dissection has sufficed to prove, as to the termination of the vertebral column.

"According to the usually adopted classification of Geoffroy St. Hiliare, this monster is one of the genus peracephalus, from the greek *περα*, beyond or further than, being prefixed to acephalus, to mark a still greater deformity, the thoracic members being absent as well as the head. Hilaire states that about fifty cases of this form of monstrosity have been recorded, and that, from their examination, it appears that generally they are not born of women in labor for the first time; delivery takes place from the sixth to the eighth month; the gestation is double; the placenta is common to both children. The other child is well formed; the sex is the same; and the acephalic child is almost never born first. All these circumstances, said by St. Hilaire to generally occur, have been noticed in this instance, as already stated; the only exception being that in this case the first child had a club-foot, instead of being well formed.

"As, for the sake of preserving the specimen, the dissection of this monster was not carried far, the principal modifications of the internal organization found in these cases will be given as they are stated by St. Hilaire. As in every other point his statements correspond to what occurred, and was found in this case, there is no reason to doubt that they would also correspond to what would be found were the dissection completed."

The author then gives a condensed account of M. St. Hilaire's description of the internal organizations of these monsters, and winds up with some very just remarks on the science of embryogeny.—(*The American Journal of the Medical Sciences*, April, 1858, p. 370.)

*My First Case of Placenta Prœvia.* By JOHN H. GRANT, M.D., Conwayborough, South Carolina.—Dr. Grant found this patient in a very miserable condition; she told him she had been flooding, off and on, for six weeks; supposed herself seven months advanced. The case is thus described: "Upon making a per-vaginal examination, the os uteri was found sufficiently dilated to admit of the operation of turning; the head of the child occupied the cervix and os uteri; the after-birth was readily recognized, intervening between the head and cervix uteri; it extended around the whole circumference of the os, excepting a space of about fifty or sixty degrees. At some points the edge of the placenta could be felt projecting an inch or more into the vagina." Delivery was attempted by turning, but the woman resisted violently, and the operation was abandoned. She continued in this state for three days, when labor commenced, and in about sixteen hours the child was born, perfectly exsanguined, exhibiting no signs of life; no hemorrhage occurred after the expulsion of the fœtus. The doctor observes: "I have no doubt that this case would have done well under the use of ergot, and the child would have been born sooner than it was; but as the pressure of the head, and the remedial means adopted arrested the hemorrhage, I thought it best to let well alone." A week after the birth of her child this lady was attacked with phlegmasia dolens; the right extremity was first affected, and as the swelling subsided the left enlarged in the same manner, and went through the same process. "The patient slowly but perfectly recovered." —(*Charleston Medical Journal and Review*, March, 1858, p. 183.)

*Adhesion of the Placenta.* By JOHN W. JONES, M.D., Professor of Principles and Practice of Medicine in the Atlanta Medical College, Atlanta, Georgia. —In this case, the reporter, Dr. Jones, found that after the most careful and persevering manipulation, he could bring away only about a third of the placenta; finding it thus adherent, he resolved to leave its separation and expulsion to the efforts of nature; accordingly, in three days it came away, and the woman made a good recovery.

The treatment appears to have been very judicious, but as it contains no novelty, we do not report it.—(*Atlanta Medical and Surgical Journal*, March, 1858, page 386.)

*On some of the Causes of Puerperal Convulsions.* By RALPH N. ISHAM, M.D., Chicago, Illinois.—This is an interesting article on the causes of puerperal convulsions, and particularly discusses the relation of albuminuria to puerperal eclampsia. "Fifteen years ago, Dr. John C. W. Lever called the attention of the profession to the important fact, that in puerperal convulsions the urine is proved to be albuminous; since that time, by more careful analysis of cases and accumulated evidence, through the observations of Simpson, Dubois, Cazeaux, Blot, Frerichs, and many others, the coincidence of the albuminous urine with eclampsia is now fully established and undisputed; and no well-observed cases show this state of the urine to have been absent. In over fifty per cent. of fatal cases of convulsions, when autopsies have been made, undoubted Bright's disease has been found, which has led to a series of investigations by Frerichs, Braun, Litzmann, and others, who strenuously maintain the identy of uræmic intoxication in acute Bright's disease and puerperal eclampsia; among the proofs of which are the following:—

1st. The same precursory symptoms of which œdema is the most frequent.

2d. The same renal lesions are found after death in eclampsia and Bright's disease.

3d. The identity of the convulsions, and the fact that males may be subject to them in morbus Brightii.

4th. The two diseases are always accompanied by albuminuria.

5th. Bright's disease, as well as eclampsia, sometimes exists without the presence of œdema."

Dr. Isham quotes several cases to sustain the truth of these positions, but we cannot, without neglecting others, afford him more space.—(*Chicago Medical Journal*, October, 1858, p. 486.)

*Puerperal Convulsions from Gastric Irritation.* By J. LEVERGOOD, M.D., Wrightsville, Pennsylvania.—Gastric irritation, it has been conceded, sometimes will occasion puerperal convulsions, and the following case exemplifies the fact. This patient had eaten a small piece of raw turnip, which produced violent gastric uneasiness, terminating in convulsions. Upon consultation, it was determined to let the labor take its natural course, on the ground that "a meddlesome midwifery is bad." The result justified the course pursued, for in a short time the uterus contracted, and expelled a still-born male child. The convulsions continued for two days, and a slow recovery took place, a most rigid antiphlogistic treatment having been employed.—(*The North American Medico-Chirurgical Review*, March, 1858, p. 310.)

*A Case of Impregnation without Rupture of the Hymen.* By HOWARD SMITH, M.D., Professor of Materia Medica, New Orleans School of Medicine.—In this case, Professor Smith was called upon to attend a lady in her first confinement, whose husband assured him that there was some obstruction in the vagina; an examination was attempted, but the parts were so sensitive that the operation was an impossibility. Dr. Smith then put the patient fully under the use of chloroform, by which means he contrived to make a vaginal exploration, and ascertained that the only obstruction which existed was an unusually dense hymen.

Her labor came on naturally, but slowly, as was to be expected in a first accouchement.

"The pain which delivered the head was very severe, and I distinctly felt the hymen rupture as the head escaped into the world; the rest of her labor presented nothing of importance. She recovered without a bad symptom, without fever, and with no tearing either of perineum or walls of the vagina."

The author is aware of the fact that he may be suspected of having mistaken the fourchette for the hymen, but such an error, he says, was impossible, for the hymen was not a narrow band, but broad, thick, and nearly closing the orifice of the vagina. Had the parts not been so tender, it would have been a simple operation to divide this membrane; but, under the circumstances of the case, it was impossible. This excessive sensitiveness appears to have been purely nervous, for "there was no inflammation of the mucous membrane, and there never had been."—*New Orleans Medical News and Hospital Gazette*, June, 1858, p. 217.)

*Abuse of the Pessary.* By J. S. WEATHERLY, M.D., Montgomery, Alabama.—Dr. Weatherly thinks that the use of the pessary has been even more abused than that of the speculum. After speaking generally of the pessary, and the irritation it often produces in the mucous membrane of the vagina, he says that the pessary, so far from curing prolapsus uteri, if such really exists, "aggravates all the symptoms."

The doctor proceeds thus: "Though the pessary, in some few cases, is no doubt a valuable adjunct in the treatment of prolapsus, I doubt very much whether it ever cures a case. When I first commenced the practice of my profession, I thought I had, or would have frequent use for this little instrument; consequently, I procured a fine assortment, and, as a matter of course, I used them as often as I thought a case would admit of one, and probably much oftener than was really necessary. But, after studying more particularly the nature of uterine and vaginal diseases, I found that their use was rarely indicated, and that I could treat the majority of cases much more satisfactorily, to myself and patients, without them. Again, I found that it was almost impossible to keep one of them adjusted in the vagina of any negro woman; their duties are such, generally, as to make it very difficult, even if they tried, to retain them in situ." Dr. Weatherly says that among higher classes of patients, whose sedentary habits predispose them to uterine derangements, prolapsus, when it exists, is treated by him more as a symptom than as a disease, per se, and says: "We can cure it by remedying the engorgement or ulceration, if it exist; not, however, by introducing a pessary, and sending our patients to bed, but by using rational means for subduing the disease, and giving tone to the relaxed tissues."—(*Atlanta Medical and Surgical Journal*, June, 1858, p. 590.)

*Complicated Case of Obstetrics.* By B. T. Smith, M.D., Central Institute, Alabama.—Mrs. —— had been in labor twelve hours, when she sent for Dr. Smith. The membranes were still entire, but on examination he detected the cord by its pulsations, and from the general character of the case felt some fear of a placental presentation. On passing his finger up a second time, the waters broke, and he thus found that the head of the child and the placenta were presenting together; fortunately, the pains were but slight, and by pressing the placenta upward he managed to retain it above the superior strait until the head took full possession, and in about forty minutes the child was born. But the great difficulty in this case arose from the fact that it was found to be impossible to bring away the placenta. The doctor describes it as follows: "I introduced my hand, and found the uterus pretty closely drawn upon the placenta; I grasped the body, and expected to have my hand and it thrust out together. But judge of my disappointment—it refused to come or even yield; I could have torn it to pieces, but I did not wish to do this. It was firmly attached to the cervix, by what seemed to me a cartilaginous adhesion." After many efforts, Dr. Smith resolved to wait an hour or two for the woman's sake, as well as for his own, for he was completely exhausted. After this, large doses of ergot and biborate of soda were administered, in hope that the strong efforts of the uterus might overcome the adhesions. In four hours, powerful efforts were produced, and during one of these throes, "amounting almost to convulsion, the adhesions gave way, and the placenta was cast out."

The nurse officiously disposed of the after-birth while the doctor was busy with his patient, and thus the nature of the adhesions could not be critically made out. The woman speedily recovered. It may be observed that the absence of hemorrhage in this case was both curious and fortunate.—(*Atlanta Medical and Surgical Journal*, June, 1858, p. 593.)

*Puerperal Apoplectic Convulsions, with Spontaneous Expulsion of the Fœtus after Death.* By Benjamin Fessenden, M.D., Plymouth, North Carolina.—In this case the child was expelled from the uterus the day after the death of the mother.—(*Medical Journal of North Carolina*, October, 1858, p. 161.)

*Case of Placenta Prævia; Central Presentation; Safe Delivery.* By Richard M. Currey, M.D., Knoxville, Tennessee, Professor of Medical Chemistry in Shelby Medical College, Nashville.—This patient was in imminent danger from profuse flooding, when the doctor, during a severe pain, perforated the placenta within half an inch of the umbilical cord, the waters escaped, and the flooding ceased. A large dose of ergot was administered, and the child was speedily born. The treatment consisted mainly in the application of cold, and keeping the woman in a recumbent position. The application of the tampon was attempted in the early stage, before the os uteri was sufficiently dilated to admit of active manipulation, but could not be effected (why is not stated.) Both mother and child (a rare circumstance in such cases) survived and did well.

Dr. Currey says that he believes the success of this case was owing to its being left to nature after the presenting placenta was ruptured.—(*Atlanta Medical and Surgical Journal*, October, 1858, p. 109.)

*Uterine Hemorrhage from Hour-glass Contraction after the Expulsion of the Placenta.* By F. A. STANLEY, M.D., Winthrop.—Dr. Stanley gives four cases of this accident, in all of which he succeeded in bringing away the placenta, and producing regular contractions of the womb by the free administration of opium, and careful dilatation of the spasmodic contraction.—(*The Maine Medical and Surgical Reporter*, October, 1858, p. 197.)

*Case of Unusually Large Development of the Human Fœtus.* By CHARLES A. BUDD, M.D., Lecturer on Obstetrics in the New York Medical College, Fellow of the New York Academy of Medicine, etc.—This child measured twenty-three inches in length, weight (without the brain, for it could not be delivered without perforating the head,) a trifle over twelve pounds.—(*American Medical Monthly*, March, 1858, p. 192.)

*Fibrous Tumor of the Uterus Complicating Labor.* Reported by J. N. KIRKPATRICK, M.D., of Randolph County, Arkansas.—In 1857 this lady was delivered safely of a living child, but after labor, the placenta being expelled, a large fleshy substance was found hanging down between her thighs when a midwife in attendance sent for a physician, who pronounced it the inverted uterus, that it was very much swelled, and that as soon as he could get the swelling reduced, "he would put it back." She continued in this state for two months, miserably emaciated, perfectly anæmic, and completely bed-ridden. The doctor and her friends gave her up as a dying person. Dr. Kirkpatrick was now sent for, and on making an examination, found the womb in its natural position, and that this fleshy substance was a large polypus. "It was as large as a full-sized cocoanut, of a conical shape, and attached to the mouth of the womb by a narrow neck or pedicle." Dr. Kirkpatrick immediately removed it in one mass, by cutting this pedicle near its attachment. It bled but very little, and weighed nearly three pounds. By means of tonics, chalybeates, nourishing diet, and moderate exercise, the patient rapidly recovered.

The necessity of a thorough examination is in this case fully exemplified.—(*St. Louis Medical and Surgical Journal*, July, 1858, p. 295.)

*Anatomy of the Placenta.* By JOHN C. DALTON, M.D., Professor of Physiology and Microscopic Anatomy in the College of Physicians and Surgeons of New York.—The object of this paper is to demonstrate, by positive evidence, some points in the anatomy of the placenta which are still in dispute. It is well worthy of attention, but an analysis would occupy too much space.—(*American Medical Monthly*, July, 1858, p. 1.)

*An Inquiry into the Modern Doctrines regarding the Frequency, Importance, Pathology, and Treatment of Abrasions, Excoriations, and Ulcerations of the Os and Cervix Uteri.* By DANIEL MCRUER, M.D., Bangor, Maine.—(*The Maine Medical and Surgical Reporter*, September, 1858, p. 163.)

*Removal of the Placenta in the Early Months of Pregnancy by Evulsion.* By O. C. GIBBS, M.D., Frewsburg, New York.—(*American Medical Monthly*, New York, December, 1858, p. 400.)

*Report of a Case of Extraction of a Living Child, by Turning, after the Death of the Mother; with Remarks.* By G. W. THORNTON, M.D., Newport, Kentucky. Read before the Newport and Covington Medical Society.—(*Cincinnati Lancet and Observer*, February, 1858, p. 65.)

In this case the child was turned and extracted forty-five minutes after the mother had ceased to breathe; it lived three weeks and two days. This result illustrates the propriety, indeed the absolute necessity on such occasions, of immediate delivery. The judicious promptitude of Dr. Thornton was highly creditable.

*Remarkable Cases in Midwifery.* By GEORGE SHEDO, M.D., Denmark, Iowa.—(*American Journal of the Medical Sciences*, October, 1858, p. 341.)

"*Meddlesome Midwifery is Bad,*"—(BLUNDELL.) A dissertation read before the New London County Medical Society. By ISAAC G. PORTER, M.D., New London, Connecticut.—This excellent paper is full of sound practical sense, and should be read not only by all young practitioners, but by a great many old ones. It treats of the abuse of the tampon in cases of threatened abortion; of unnecessary bleeding in pregnancy; of converting one presentation of the head into another; rupturing the membranes to expedite labor, when no such interference is called for; mechanical irritation of the os uteri, another means often rashly used to promote relaxation; and quotes the following passage from Churchill: "Delay in the first stage of labor involves very little, if any danger, however tedious it may be, for the stress or strain comes on parts where it can safely be borne; but delay in the second stage is very different. The nervous shock is never in proportion to the first stage, but to the second." Dr. Porter explains that these different manipulations may, under circumstances, all be useful, and closes his dissertation with the following remark: "Omitting, for want of time, the discussion of other topics involving meddlesome interference in practice, such as the use of forceps and of ergot, retained placenta, and placental presentations, we only add, in conclusion, that in the foregoing remarks we have advised the *cautious use* of the tampon in abortion, not, of course, its disuse; we have spoken of *unnecessary* venesection in pregnancy, not denying that it is often proper; we have dissuaded from unnecessarily converting one presentation into another, not affirming but that it may be imperiously demanded in some cases. The same is true in regard to rupturing the membranes, to the management of nates presentations, and to the excited dilatation of the os uteri. It is not the *use* of measures, (often proper,) that we have objected to, but the *abuse*. Knowledge alone can guide us when and how to act."—(*Ibid.*, p. 345.)

*Midwifery Cases.* By RICHARD MCSHERRY, M.D., Baltimore.—This article comprises two cases: one of cancer of the womb, with unyielding os uteri, in which the bones of the head were removed in detached pieces; the body was brought down without much trouble; child putrid. The other case is one of spontaneous hemorrhage in a new-born infant, resulting in death. The doctor was called to see it a few hours after birth, and found it dead. Upon turning it on its face, there flowed from its mouth a discharge of thin blood, or bloody serum. In considering the cause of this hemorrhage, Dr. McSherry attributes it to the condition of the mother, who had lived for upwards of two years in an enervating climate, at a barren and desolate outpost, (her husband was an officer,) where the troops, officers and men, subsisted on the army rations; the food was too coarse for her, and she lived almost exclusively on bread and butter, and water, and none of these of the best. "Viewing all the facts, I considered that the child died of scurvy; and in fact, upon inquiry, I found that both officers and men had experienced something of that disease at the same station."—(*Ibid.*, p. 352.)

*Placenta Prœvia.* By J. P. TERRELL, M.D., Illinois.—The placenta in this case presented directly over the os uteri, which was dilated only to the size of a fifty-cent piece, and the flooding so continuous and alarming, that the doctor resolved to separate the placenta entirely from the womb; this he accomplished, at the same time rupturing the membranes. The result was fortunate; the pains came on with vigor, the placenta was pushed on one side, as much as possible, and the child's head became rapidly engaged in the orifice of the womb, and (no doubt by the pressure it exerted) stopped the hemorrhage. The woman continued all night in labor, the continued rigidity of the os causing delay; fortunately, in the course of the day it relaxed; the hemorrhage, still more fortunately, did not return, and near twelve o'clock P.M., with the aid of the forceps, she was delivered. The child was dead, (as might be expected, after such severe flooding,) but the mother recovered, and is now enjoying excellent health.—(*The Chicago Medical Journal*, July, 1858, p. 297.)

*Cases of Uterine Disease.* Read before the Boston Society for Medical Observation, and communicated for the *Boston Medical and Surgical Journal.* By FRANCIS MINOT, M.D.—Three cases are here given to illustrate two different uterine diseases. "The first is one of constriction of the mouth of the womb and of the canal of its neck, accompanied by dysmenorrhœa, for which dilatation was performed, followed by considerable relief to the symptoms. The last two are examples of granular disease of the mucous membrane of the os uteri. One of these patients had prolapsus uteri, leucorrhœa, and the train of general symptoms usually accompanying that condition; she was entirely restored after a course of treatment consisting chiefly of astringent applications to the cervix, and continued for upwards of three months. The principal symptom presented by the other patient was menorrhagia; the case is adduced chiefly to show the advantage derived from the early treatment of the disease."—(*The Boston Medical and Surgical Journal*, September, 1858, p. 9.)

*An Essay on the Physiology of Menstruation.* By EBEN HILLYER, M.D., Rome, Georgia. Read before the Medical Society of the State of Georgia, at the annual meeting in Madison, and ordered to be printed, April, 1858.—(*Southern Medical and Surgical Journal*, December, 1858, p. 798.)

*Puerperal Fever.* By J. A. WINDLE, M.D., Blountsville, Indiana.—(*Cincinnati Lancet and Observer*, August, 1858, p. 460.)

*Remarks on the Employment of Pessaries, with the Description of a New Instrument* (*with illustrations.*) By E. NOEGGERATH, M.D., New York.—That pessaries have by mismanagement, and their application to inappropriate cases, frequently done mischief and complicated the evils they were intended to counteract, is a fact which I believe few physicians who have seen much practice will deny; at the same time it is no less generally admitted that there are cases in which the pessary is not only of service, but actually necessary to a cure. Dr. Noeggerath is decidedly of this opinion, and before describing the new instrument recommended by him, thus points out the cases which he considers as imperatively demanding the use of the pessary:—

"1st. Prolapsus in that portion of society, where it is most commonly met with, viz., in poor women, who have neither time nor means to undergo a complicated course of medical treatment.

"2d. Prolapsus of a very long standing, where the causes may be removed by careful management, while their effect upon the falling of the womb, resists every other means.

"3d. Those cases of prolapsus, not unfrequently seen even in otherwise strong and healthy women, where the most thorough examination is unable to detect anything apart from the normal state besides a relaxation of all the apparatus supporting the womb. Admitting that a pessary is required in a given case, the important question occurs, what kind is to be chosen? The requisites of a good instrument are as follows: first, it must retain the womb in, or near its natural position; second, it must neither irritate the womb nor the vagina; third, it must not interfere with the patient's moving round, sitting, or the excretion of the urine or fæces; fourth, it must be composed of a substance which resists the corrosive influence of the secretions from the genitals; fifth, it must be so constructed as to be easily introduced and removed by the patient herself; sixth, it must be as cheap as possible."

Having thus, as it were, introduced the describer, our space will only admit of our giving a brief account of the nature of the instrument.

"Dr. Zwank, of Hamburg, published an instrument in 1853, which he called a hysterophor. It consists of two ovoid thin pieces of metal, covered with India-rubber or of wood, connected on one end by a joint; in the neighborhood of this joint, on the external sides of the wings, is a metallic pin on each side, two inches long, which can be screwed together at the lower end. (Figures are given in the original paper, to assist the reader in comprehending the mechanism of the instrument.) In applying the instrument, the wings are approached as much as possible, and introduced so that its convex portion is turned toward the sacrum and pushed upward as high as possible, toward the anterior portion of the laquear vaginæ, in front of the cervix uteri. Afterwards the lower ends of the metallic handles are compressed, and fastened by the screw. (See Figure 3 in the original.) In this position the instrument is retained by itself.

"About the same time, Dr. Schilling, of Munich, invented quite a similar instrument to that of Zwank, the only difference being that the movement of the wings is effected and can be regulated by the screw at the lower end."

The author states that these instruments are superior in every way to any yet invented, but still they have some disadvantages owing to the substance of which they are composed. "The greater number of them as now in use being covered with a coat of vulcanized India-rubber, the discharges of the vagina destroy it in a very short time. After this has been done, the metallic portions begin to rust and decay, thus irritating the vulva; the furrows of the screw at the lower end begin to crust, or the screw, if turned too firmly, cannot be untwisted. In order to avoid these inconveniences, Dr. Eulenburgh, of Coblenz, modified Dr. Zwank's pessary, and described his instrument in a short thesis in 1857. It is made entirely of boxwood, and its wings are a little differently shaped, viz.: they are slightly curved downward at both ends, so that the lower side forms a concave surface. In consequence of this shape, the lateral branches closely adapt themselves to the inner surface of the descending ramus of the pubis, thus presenting a kind of hook, which gives a strong hold to the instrument when in the vagina. Both wings move in the centre part by two joints, thus leaving a hole in the middle, through which the secretions of the vagina are allowed to escape. Instead of the screw, Dr. Eulenburgh perfected the opening and shutting of the wings, by means of an elastic India-rubber ring, which runs in a channel around the body of the hysterophor immediately below the two joints. (See Figures 4 and 5 in the original.) As every particle of metal is avoided, (except the small pin running through the joint,) and as the boxwood resists more than any other substance the corrosive influence of the vaginal discharges, it is lighter, will keep longer, and will cause less irritation than the other instruments.

"For my own part, I avoid the use of pessaries as much as possible. But I have under my care a number of cases, in which a pessary was the only remedial means justifiable. I have tried a great variety of them, and have now come to the conclusion, that Zwank's (or Eulenburgh's) hysterophor answers better the requisites of a good pessary than any other." The author asks practitioners to give it a fair trial.—(*The New York Journal of Medicine*, September, 1858, p. 198.)

*Uterine Disease.* By W. H. MILLER, M.D., Henderson, Kentucky.—Dr. Miller gives three cases of uterine disease, in which the lunar caustic was applied locally with great benefit. The symptoms of the three cases are very much alike, viz.: paleness, poverty of blood, capriciousness of appetite, constant pain in the back, burning pains in the groins, leucorrhœa, etc. All the cases were treated very much upon the same plan.

First case: Examination with the speculum discovered inflammation and ulceration; treated with nitrate of silver to the neck of the womb inside and out; internally, she was put upon the use of iron, tonics, etc.; was perfectly relieved; afterwards became pregnant, and was delivered of a healthy child at full term.

Second case was that of a very delicate woman, predisposed to consumption. Examination discovered abrasion and ulceration of the os uteri, which was cauterized five times, at intervals of five or six days; absolute rest was enjoined, tonics prescribed, along with emollient injections of flaxseed. "This lady has since conceived, but is not yet delivered."

The third patient added to ulceration and inflammation, retroversion of the uterus. "Being satisfied that the retroversion was owing to the congestion of the body and the consequent increase in its weight, and having been taught that the best plan to cure it was to cure the inflammation, I scarified both lips freely, and cauterized the urinal canal; enjoined absolute rest, mild unirritating diet, emollient injections, and a tonic, consisting of infusion of colombo, of the U. S. Dispensatory, one ounce three times a day. In giving a history of these three cases, I do not suppose that I am communicating anything new, but only wish to bear the weight of my testimony and experience, limited as it is, to the value of the local treatment of these cases."—(*Nashville Journal of Medicine and Surgery*, September, 1858, p. 197.)

*A New Uterine Elevator.* By J. MARION SIMS, M.D., Surgeon to the Woman's Hospital, New York.—This (as stated by the author) is an improvement of Simpson's Sound, by making a joint or hinge in it near enough the end to prevent its striking against the fundus. In Dr. Simpson's hands, we do not question the safety of his instrument, but as a weapon for general use, we have always thought it somewhat dangerous. Dr. Sims describes his improvement, and illustrates with a wood-cut; it is worthy of attentive consideration.—(*The American Journal of the Medical Sciences*, January, 1858, p. 132.)

*Cupping the Interior of the Uterus.* By HORATIO R. STORER, M.D. Read before the Boston Society for Medical Observation, October 19, 1857.—This operation was performed by Dr. Storer, in two cases of amenorrhœa, for which every other means had been tried, and had failed. In both cases, the uterus was found under-sized and undeveloped.

In neither, however, was there any obstacle to the flow and free excretion from the uterine tissue. The author says: "They may each require an extension of the treatment, but its immediate and decided effect for good, both on the uterine system and general health, give hope that the improvement may prove permanent."

This operation was first proposed and apparently first practiced by Dr. Simpson, of Edinburgh, but we do not think that accounts have yet been published; therefore its success and safety remain to be proved. The author describes his instrument, which is a modification of Simpson's. He makes the following remarks: "I have used the cup in other cases than those reported, in every instance with the effect of reducing the sanguineous discharge, and I have never seen from it any permanently disagreeable result. Its power upon the nervous system is, however, at times so decided, that its use should be with care. In one case where the introduction of the instrument had been painless, the first play of the piston occasioned instant and alarmingly profound syncope."—(*The American Journal of the Medical Sciences*, January, 1858, p. 117.)

*Ulceration of the Os Uteri.* Read before the Cook County Medical Society, Chicago. By J. N. GRAHAM, M.D.—This is a good paper, calling the attention of the profession to the great discrepancy in the different hospital reports of this malady, the difference of opinion as to the extent of the disease, and the treatment best adapted to its cure. "Statistics have been piled up before us to fortify whatever position was assumed or deemed to be right, without regard to the source from whence the material was taken."—(*The Chicago Medical Journal*, February, 1858, p. 70.)

*Advantages of the Prone Position in examining the Fœtal Circulation as a Diagnostic Sign of Pregnancy.* By W. BYFORD, M.D., Professor of Obstetrics in Rush Medical College.—"The fœtal heart," says Professor Byford, "may be heard to beat several weeks sooner by placing the woman on her face, with the abdomen pendent, than in the supine or erect position. In the prone position, with head lower down than the level of the feet, and the abdomen hanging freely down from the spinal column, the uterus rises out of the pelvis, and by its weight, keeps in close proximity to the anterior wall of the abdomen; and the fœtus being of greater specific gravity than the liquor amnii, will settle down on the most dependent part of the uterus. In this condition of things, the ear of the observer, or the end of the stethoscope, is placed against the lowest part of the uterine tumor, and pressed pretty firmly upward against it, and thus brought pretty close to the heart of the contained fœtus, nearer, in fact, than in any other position that can be assumed."

Would there not be some difficulty and inconvenience in placing the woman in such a position? We have a misgiving that the greater number of ladies of our acquaintance would object.—(*The Chicago Medical Journal*, February, 1858, p. 77.)

*A Few Remarks upon Uterine Hemorrhage.* By O. C. GIBBS, M.D., Frewsburg, New York.—The remarks in this paper are entirely confined to the treatment of passive uterine hemorrhage, apparently resulting from transudation of blood from the uterine walls in consequence of passive congestion and relaxation of fibre. Dr. Gibbs justly observes, such cases are often cured by the use of ergot, muriated tincture of iron, and astringent uterine injections; but in some cases of uterine hemorrhage, apparently resulting from some unnatural condition of the lining membrane of the uterus, Dr. Gibbs has found injections of tincture of iodine into the uterus, conjointly with the internal administration of iron and quinine, to work speedy cures.

"The tincture should be reduced with an equal quantity of water, to which a little iodide of potassium is added to prevent a deposit of pure iodine, which will sometimes result on the addition of water. The injection should be used every second or third day, two or three drachms being used at a time."

Dr. Henry Savage, of London, has reported two or three cases of menorrhagia cured by iodine injections, other means having been tried without effect.—(*The Cincinnati Lancet and Observer*, April, 1858, p. 213.)

*Milk and its Adulterations.* A paper read before the Cincinnati Academy of Medicine. By W. T. BROWN, M.D.—This paper has evidently been very carefully prepared, and contains much useful information with regard to the constituent elements of milk, and the effect produced upon these elements, to the destruction of their nutritious properties, by feeding cows upon distillery slops.—(*The Cincinnati Lancet and Observer*, October, 1858, p. 577.)

*Case of Traumatic Occlusion of the Vagina.* Read before the Cook County Medical Society, at its regular meeting, April, 1858. By ERNST SCHMIDT, M.D. The subject of this paper was a poor woman, who, in her first labor, became the victim of both mismanagement and neglect; the consequence was such a degree of laceration and destruction of the mucous membrane of the vagina that perfect occlusion took place; the menses were retained, and at every monthly period her sufferings were excruciating. Doctors, quacks, and midwives were called in, who, without making any examination, administered emmenagogues of the strongest kind, both internally and externally; her sufferings of course increased. At length, Drs. Schmidt and Wagner saw her, and having, by proper exploration, ascertained the nature of the case, determined to operate. The operation is described as follows:—

"The patient was laid across the bed in a position similar to that for the operation of lithotomy, and then anæsthetized. Dr. Wagner stood at her left side, the index finger of his left hand in the anus, and with the right hand holding a silver catheter lying in the bladder. I knelt down between her legs, brought the index of my left hand into the vagina like a probe, upon which I introduced a very fine-pointed and somewhat crooked tenotome with the edge upon the concave side, at an equal distance from the rectum and the catheter; I penetrated the vagina, and pressed the knife vigorously forward, about half an inch deep, till I felt that the resistance was yielding; then I dilated the opening externally by an incision about half an inch long, whereupon thick tarry blood commenced flowing out, and in this way indicated that the first success was attained."

This operation was successful; the woman recovered, is now in good health, and menstruates regularly. Dr. Schmidt expresses some degree of regret "that the constriction was not opened and widened so far as to lodge the cervix uteri in it as in another fundus vaginæ, and to cause a fastening of the cervix with the surface of the incision by adhesive inflammation;" but adds, he fears that such a proceeding would have produced a fatal peritonitis. Indeed, from the account of the case, it appears that the opening of the adhesions, so as to reach the os uteri and afford an egress to the incarcerated menstrual fluid, had nearly proved fatal. Should the woman become again pregnant, the important question arises, what will be the result? We should be inclined to fear, either a free incision of the vaginal stricture or dangerous laceration.—(*The Chicago Medical Journal*, May, 1858, p. 197.

*Criminal Abortion.*—In this journal, there is a full history of the movement which has lately taken place among the profession, both in Massachusetts and New Hampshire, for the purpose of checking, if possible, the continual perpetration of criminal abortion. It appears from the report, that many respectable people cannot realize the wickedness of this act, but have recourse to it as a convenient family arrangement. The following paragraph, which we quote from the report, is particularly refreshing: "We have in this city a woman engaged in the nefarious occupation of producing abortion, with whom our authorities are unwilling to meddle, on account of her popularity as a *devoutly pious* person."

This extract is from a journal of 1857. We have in every other case confined ourselves strictly to reporting for 1858; but there really is something so original and grotesque in the devoutly pious woman who lives by a systematic course of child murder, that we could not make up our mind to exclude it. It seems that piety, as well as charity, covereth a multitude of sins.—(*New Hampshire Journal of Medicine*, Manchester, July, 1857, p. 208.)

*Description of a New Midwifery Forceps, having a Sliding Pivot to Prevent Compression of the Fœtal Head, with Cases.* By GEORGE T. ELLIOT, JR., M.D.—The inventor of this forceps recommends it as a great improvement; and though we never ourselves found that compression or injury to the child's head was by any means a necessary consequence of the application of any well-constructed forceps, yet we have no doubt, that cases may arise in which Dr. Elliot's forceps might prove very useful. The doctor gives a full description of the instrument—length, breadth, thickness, etc., with illustrative woodcuts. We have no intention to describe the instrument, but refer our readers to the original paper.

To illustrate the immense power which may in some cases be required for delivery, Dr. Elliot gives us the following graphic account:—

"Called in consultation with Dr. Freeman, to Mrs. M., a twin, whose sister had died after her second difficult labor; this one, a primipara, twenty-six years of age, at full term, and had been in hard labor for eighteen hours. Second stage—os fully dilatable, soft parts cool, head presenting the second of Nægele, right occip. post., and wedged in superior straight after commencing rotation; no fœtal heart appreciable, but strong uterine soufflé; Simpson's forceps preceded by chloroform and baptism; tremendous tractions, until one blade straightened completely. Another pair; no better success. Perforator and crotchet now used; hard work. Finally replaced the forceps around the diminished head, and effected its delivery; tight work even with the breech."

This feat diminished the child's head; has it not annihilated Simpson's? We much fear it, and at the same time candidly admit, that we cannot altogether avoid the sin of envying the operator the possession of such heroic muscular force; two pairs of forceps straightened out!! Was there in this case no danger of *straightening out the patient?*—(*The New York Journal of Medicine*, September, 1858, p. 160.)

*Case of a Mole, or False Conception Retained in Utero.* By SAMUEL B. CLARK, M.D., Brothersville, Georgia.—This case is interesting, from the fact that the mole was retained in utero for full nine months; a very unusual circumstance.—(*Southern Medical and Surgical Journal*, August, 1858, p. 563.)

*Obstetric Medicine.* By W. P. MOORE, M.D., Linwood. Read before the Tennessee State Medical Society, April, 1858.—We have little space remaining, and therefore cannot give even a condensed statement of the contents of this paper. The following short paragraph we quote, because we think it full of sense and truth: "The authors on obstetricy are as varied in their articles as the cases with which we meet, and amid a description of the instruments which are thrown among us, the physician who forms his practice upon the great principles of medicine, and who is guided by the disclosures of nature, as the indications of the case, is astonished when he reads of the frequent use of the perforator, the difficulties in turning or the application of the forceps."—(*Nashville Journal of Medicine and Surgery*, July, 1858, p. 6.)

*Bone in an Ovarian Tumor.*—Dr. Woodward exhibited to the Pathological Society of Philadelphia some pieces of bone from a large ovarian cyst. The ovary in this case contained an extensive deposit of encephaloid, and the cyst contained also hair and fat.—(*North American Medico-Chirurgical Review*, January, 1858.)

*Obstetrical and Medical Cases.* By GEORGE T. ELLIOT, JR., M.D., Physician to Bellevue Hospital, the Nursery and Child's Hospital, and the Lying-in Asylum.—This is a long report of the different cases treated by Dr. Elliot in the Bellevue Hospital, and contains a great deal of interesting and useful matter.—(*The New York Journal of Medicine*, July, 1858, p. 37.)

*A Case of Difficult Labor; the Child weighing Sixteen Pounds.* By F. S. JAQUETT, M.D., Philadelphia.—This, we take it, is the largest new-born child recorded in the annals of Philadelphia obstetricy. Ramsbotham is stated to have delivered a woman of a child weighing sixteen and a half pounds avoirdupois, and Mr. Bloxam, in the *London Lancet*, describes a case where he brought a child into the world with the forceps, which weighed seventeen pounds and twelve ounces, and whose length was twenty-four inches; this last is the largest on record, that is, so far as we have ever been able to ascertain.

In Dr. Jaquett's case, the labor had continued many hours, but the head remained immovable at the superior strait; at length, satisfied that instrumental aid was necessary, he applied Davis's short forceps without difficulty; but upon making strong traction, they slipped continually; he then sent for Professor D. Gilbert, who employed a modification of Dr. Bethel's forceps. After considerable difficulty, the child was brought into the world; it was still-born. Astonished by its great size, the doctors immediately proceeded to weigh it, and found its weight to be sixteen pounds.

The contractile power of the uterus being apparently exhausted, the patient showed a strong disposition to hemorrhage, but by means of friction and the administration of ergot, this difficulty passed away, and she recovered without any bad symptom.—(*Philadelphia Medical and Surgical Reporter*, September, 1858, p. 571.)

*Puerperal Fever.* By J. A. WINDLE, M.D., Blountsville, Indiana.—Dr. Windle, after some few remarks upon the doctrines of Gordon, Hays, and others, and the teachings of Dr. Meigs, whose exclusive views of the pathology of this fatal disease makes the sheet-anchor in the treatment of every case the prompt and decided use of the lancet, proceeds to say, that though he perfectly agrees with the learned Philadelphia professor, in his view of the pathology of the disease, yet he thinks that there are many other means that may be employed which are more efficacious than blood-letting, in point of curative power.

Dr. Windle's position is, that according to his experience the disease is partially, if not exclusively dependent upon a malarial influence. He infers that this must have been the case in the particular epidemic, which he had an extensive opportunity of studying, from the fact that the remedies most successfully used by him were of an antimalarial nature. We quote the following passage from the doctor's paper:—

"Professor T. D. Mitchell, in his work on Therapeutics and Materia Medica, asserts that the great antiperiodic, sulphate of quinia, has been successfully employed as a prophylactic against its access. Now as the disease has been prevailing for nearly a year in our section of the country, and so much pain and suffering, together with the loss of some lives, was induced thereby, that I thought it my duty, as a philanthropist, to use some prevention, if possible, to stay its ravages."

In short, the author used the sulphate of quinia largely as a prophylactic, and found the effect highly satisfactory. Thus, in twenty cases to whom the quinia was administered in this way, not one was attacked by the disease; while among an equal number not submitted to the prophylactic treatment, eighteen suffered more or less severely from puerperal fever.

Dr. Windle recommends this use of the sulphate of quinia to the attention of the profession, for though his experiments were apparently satisfactory, yet he feels that a much wider field of trial is necessary to establish the efficacy of the plan.—(*The Cincinnati Lancet and Observer*, August, 1858, p. 460.)

*Puerperal Fever.* An abstract of the discussion at the Paris Academy of Medicine. Collated from the French journals for the *American Medical Monthly*, July, 1858, p. 19.—This is a most interesting paper, but to make it intelligible to the reader would require more space than can be afforded in an ordinary report; we therefore refer those interested in the subject to the original article.

*Sulphate of Zinc in Hemorrhages.* By WILLIAM HAUSER, M.D., Speir's Turnout, Georgia.—Dr. Hauser has used for many years the sulphate of zinc in hemorrhages, and states that in his hands he has never known it fail to arrest the most obstinate bleeding. He throws a teaspoonful of the salt into a teacup half full of cold water, and it is ready for use.

In the treatment of obstinate epistaxis he throws this solution up the nostril by means of a syringe, and assures us that the bleeding capillaries are immediately closed, and all danger ceases.

In uterine hemorrhage, when other modes of treatment fail, he uses the same solution, but adds, that he hesitates long, on account of the pain it is sure to produce when applied to a bleeding mucous surface.—(*Atlanta Medical and Surgical Journal*, November, 1858, p. 135.)

*Catamenia during Pregnancy.* By J. F. DURGIN, M.D., Portland, Maine.—This is a very singular and interesting case. It appears that this lady, at the fourth month of her pregnancy, was taken with violent convulsions, which, after some hours' continuance, terminated in violent uterine pain and a somewhat profuse discharge of blood, but no miscarriage.

The following is Dr. Durgin's account of this singular phenomenon: "When I first saw her, before making an examination per vaginam, I supposed the case might result in miscarriage, though I felt sure of the nature of the exciting cause, believing, from representations of the patient and her nurse, that her pains had been so severe, and her flowing so free, as to endanger the life of the fœtus, and render its expulsion certain. With this impression, I waited awhile to watch the character of the pains, which recurred about every ten or fifteen minutes generally, though not always accompanied with convulsions, more or less severe. A few intervals having passed, in the midst of a pain I made an examination with the index finger, and ascertained that the os was not dilated, and that the pains did not present that expulsive tendency which I should expect in a case of threatened miscarriage. Suspecting from this that it might be only a hysteric monthly excitation, attended with menstrual flux, on further investigation I learned that she had been subject at times to similar nervous convulsions, but not so often as monthly, and that the character and quantity of the present flow were very nearly like her usual courses, differing, if at all, in its greater abundance at this stage of the menstrual term. Accordingly I ordered a mixture of tinc. valerian and assafœtida, equal parts, with directions that a teaspoonful should be given every hour till she became quiet. The following day I found my patient somewhat better, though the flowing still continued, but attended by less severe pain, and the convulsions recurring at more protracted intervals. In a few days all the symptoms subsided, till the following month, November, when they again returned in all respects as before."

In short, these same symptoms, viz., convulsions, pain, and hemorrhage, recurred regularly every month, from the fourth month of utero gestation until full term, when the true pains of labor came on, with slight convulsions, which, upon the abstraction of fourteen ounces of blood, disappeared, and the lady was delivered of a fine healthy male child without difficulty or danger. In less than two weeks the patient was walking about the house, attending to her customary duties.—(*Nashville Journal of Medicine and Surgery*, January, 1858, p. 42.)

*Tartar Emetic in Obstetrics.* By J. W. Crooks, M.D., Rockport, Indiana.—The use of tartar emetic in cases of rigid os uteri has, from our earliest recollection, been recommended by obstetrical practitioners; we have used it ourselves, and thought that it was sometimes useful. Dr. Crooks speaks very highly of its powers; indeed, he is perfectly enthusiastic upon the subject, and seems to labor under the delusion, so common among medical authors, that his recommendation is a novelty.—(*Nashville Journal of Medicine and Surgery*, May, 1858, p. 389.)

*Ointment of Iodine and Belladonna in Inflamed Mammæ.* By J. S. Weatherly, M.D., Montgomery, Alabama.—Dr. Weatherly has used the following recipe with admirable effect in several severe cases of inflammation of the mammæ:—

℞.—Iodinii, grs. xv;
Ext. Belladonnæ, grs. xx;
Cerati, ʒij. Mix.

(*Atlanta Medical and Surgical Journal*, September, 1858, p. 6.)

*Inflammation, Ulceration, and Induration of the Os and Cervix Uteri, and Use of the Speculum.* By W. M. Chambers, M.D., Charleston.—This article is a defence of the use of the speculum, and the application of remedies directly to the neck of the womb for the above-named diseased conditions. The author seems to have had considerable experience, and is well informed on all that has been written on the subject. He gives particular directions for the manner in which the speculum (evidently a favorite instrument) should be introduced, and the medical applications made, which will no doubt be useful to many young gentlemen who are ambitious in the speculum way. We confess that we have no prejudice against the use of the speculum, if its application be necessary; but at the same time think that some of its advocates have, to the dishonor of the profession, disgracefully abused its use. The truth is, that it is an operation easily performed, and, unfortunately for its reputation, has hence become a fertile source of profit to ignorant quacks and charlatans of every description. In the hands of educated and conscientious men, we do not doubt its usefulness.—(*The Chicago Medical Journal*, September, 1858, p. 451.)

*A case of Puerperal Convulsions.* By W. S. Findlay, M.D., Tazewell, Claiborne County, Tennessee.—This was an ordinary case of puerperal convulsions, in which the lady was delivered by Dr. Findlay with the aid of the forceps. The treatment consisted in delivering as speedily as possible, blood-letting, and the use of purgatives and nervous stimulants. The patient recovered perfectly.—(*The Medical and Surgical Reporter*, March, 1858, p. 154.)

*Case of Occlusion of the Vagina.* By John Bozzell, M.D., Cape Elizabeth. It appears that a complete occlusion of the vagina had in this case taken place, from injuries received during a previous difficult and protracted labor, which could only be terminated by the operation of craniotomy. The occluded vagina was partially opened by Dr. Bozzell with the knife, and the curative treatment pursued by a dilating process. This was diligently followed for the space of six months ; at the end of which time, the diameter of the canal was not more than three lines. "Menstruation was regular, and the patient, whose health in other respects was good, becoming weary, further efforts were abandoned. She was provided with a metallic tube to keep the canal open, kept in place by a T-bandage."

At length, at the suggestion of Dr. Tewksbury, of Portland, the reporter tells us that he commenced the use of the elm-tents. "They worked admirably. At the end of three months I was able to introduce one an inch and a half in diameter."

In consequence of this state of things, the lady became pregnant, the doctor was alarmed for the safety of his patient, but resolved to let gestation go on, in the mean time pursuing the dilatation with great perseverance. Finding, at the end of the seventh month, that the vagina would admit the introduction of a ball three inches in diameter, he resolved to bring on premature labor. The following quotation shows the wisdom of this decision :—

"On the 25th of February last, this being the beginning of the eighth month, I threw oz. viij. of warm water into the uterus, to separate the membranes, and thereby induce premature labor. This I repeated on the 26th, 27th, and 29th, when the pains became regular, and labor fairly set in. At four o'clock P.M., the os uteri was dilated to the size of a Spanish dollar, but being quite rigid I took from the arm oz. xij. of blood, and gave ol. ricini oz. i. This operated in two hours, after which the pains increased in force and frequency, causing the membranes to rupture. The head did not escape from the womb till midnight. It was now fairly in the vagina, and bearing hard upon the strictured portion, which gradually yielded, and though emphatically a tight fit, the child finally escaped at two o'clock on March 11th.

"To-day, March 30th, both mother and child are doing well; weight of child five and a half pounds."—(*The Maine Medical and Surgical Reporter*, June, 1858, p. 12.)

*Observations on the Identity of Erysipelas and Puerperal Fever; Diffuse Inflammation Consequent on Erysipelas; Poisoning of the Blood after Parturition.* By JOHN O'REILLY, M.D., Licentiate and Fellow of the Royal College of Surgeons, Ireland, Resident Fellow of the New York Academy of Medicine.—The ever-beginning and never-ending subject of puerperal fever we cannot pretend to enter into in a report like this. Dr. O'Reilly's article is a good one, and we recommend its perusal to the medical public; it will be found in the *American Medical Gazette*, December, 1858, p. 706.

*Case of Cancroid Ulcer of the Os Uteri; Excision of the Entire Cervix; Recovery.*—By C. E. ISAACS, M.D., Consulting Surgeon to King's County and Blackwell's Island Hospitals. After a consultation with several eminent New York physicians, this ulcer was pronounced malignant. Dr. Isaacs touched it several times with the actual cautery, with no permanent good effect; he then determined to remove with the knife the whole neck of the uterus; accordingly, the operation was performed. It is fully described in the article before us, and the result, so far as is at present known, promises a perfect cure.—(*The New York Journal of Medicine*, January, 1858, p. 46.)

*Cases of Puerperal Convulsions, with Remarks upon the Treatment of Eclampsia.* By E. N. CHAPMAN, M.D., Physician to the Long Island College Hospital, Brooklyn, New York.—This article opens with some general remarks upon the nature of puerperal convulsions, in the course of which the author gives it as his opinion, that certain electrical conditions of the atmosphere often act as predisposing causes. He points out the distinction generally admitted between hysteric, epileptic, and apoplectic convulsions, and gives some statistical statements from Churchill, showing that 79 cases of convulsions occurred in 38,306 cases of labor, or 1 in about 485. In Dublin, 30 cases in 1600, or 1 in 58 labors. From the year 1829 to 1842, at the Maternité, Paris, at the Clinique of Accouchements, the disease occurred 10 in 12,500 labors, or 1 in 1250 cases. Here is a difference so vast, that it seems perfectly unaccountable—we might almost say incredible. Dr. Chapman suggests a colder climate and frequent electrical changes of the atmosphere as a reason for its greater frequency in England and Ireland; but if sudden electrical changes have so much influence in producing puerperal convulsions, the women of the United States would be more liable to their attack than even those of England and Ireland, which is certainly not the case.

In speaking of the etiology of the affection, Dr. Chapman makes the following observations:—

"The etiology of this terrible malady is left in great obscurity by writers on this subject, and they make their statement of causes in a very loose manner. Evidently they know but little definitely on that point beyond vague generalities, affording no fixed data on which to found a rational treatment. How, I would ask, are we to benefit our patient, when we are ignorant of the very essence of the disease, and are driven to the fearful necessity of fighting an enemy in the dark? The causes suggested are atmospheric or endemic; the puerperal condition; excentric, as sympathy with the stomach, bowels, or uterus; mental emotions, fright, difficult and tedious labor; causing an accumulation of blood in the head, and pressure in the sacral nerves, etc. etc. First causes are not reached in this catalogue, and we must, if we look no deeper, be satisfied with some trivial exciting cause."

We are somewhat surprised to find that the author, in the midst of his murmurs over our want of definite knowledge of the causes of puerperal convulsions, should have entirely ignored the subject of albuminuria, a predisposing cause, which of late has so prominently occupied the attention of the profession. But Dr. Chapman's avowed object in publishing this article is to describe several cases of puerperal convulsions which occurred under his own immediate observation. They are nine in number, two of which died very soon after seizure, without much treatment of any description whatever; these the doctor calls apoplectic cases.

The other seven are epileptiform, two of which died, and five recovered. These cases are interestingly described; but as the original report can be easily obtained by all who are interested in the subject, we do not think it necessary to quote particulars.

On the subject of treatment, Dr. Chapman enters somewhat fully, particularly recommending the use of antimony as an auxiliary to the lancet, in cases which are accompanied by congestion of the brain. He says:—

"I consider that the antimony has a double action: the one, sedative and antiphlogistic; the other, antiphlogistic and contra-stimulant; and that from these combined effects it has great power in diminishing the capillary circulation. On this principle I would explain its action in congestion and inflammation of the lungs. It appears to me to exercise the same power in head affections, for the same reasons. To produce favorable results, the antimony must be given at an early stage, before the congestion is permanent. This remedy is particularly adapted to those cases in which there is great turgescence of the head, and generally free doses will be tolerated. In the opposite condition of the brain, vomiting will be apt to occur from the smallest quantity; and it may not exercise the least power over the disease. In such a condition, opium is our reliance."

This author enters his protest against the use of chloroform in puerperal convulsions, and strongly enforces the rule, to empty the womb of its contents by any justifiable procedure, without delaying to bleed, or wasting time in any preliminary treatment. "After the child is born, we can meet the demands of the case with better hopes of success."—(*The New York Journal of Medicine*, November, 1858, p. 345.)

*Parturient Effects of the Sulphate of Morphia.* By H. L. BYRD, A.M., M.D., Professor of the Principles and Practice of Medicine in Oglethorpe Medical College, Savannah.—Dr. Byrd in this paper states it as a therapeutical fact, which has been repeatedly demonstrated under his own observation, that sulphate of morphia increases the parturient efforts of the uterus. We quote the following passage to show the strength of his conviction on this subject:—

"I do not regard morphia as possessing superior properties to the secale cornutum, but I believe it is as generally certain in its effects as that article; indeed, I have several times administered the morphia with success in arousing the dormant contractions of the uterus, where a genuine article of the secale had failed in increasing or rendering more persistent the uterine contractions."—(*Oglethorpe Medical and Surgical Journal*, June, 1858, p. 73.)

*A Valuable Remedy for Dysmenorrhœa and consequent Sterility.* By E. D. FENNER, Professor of Theory and Practice, New Orleans School of Medicine.—Dr. Fenner recommends the following prescription for the relief and cure of dysmenorrhœa:—

| | |
|---|---|
| ℞.—G. Guiaci. | ℥j |
| Bals. Canadens. | ℥j |
| Ol. Sassafras. | ʒij |
| Hydrarg. Chlor. Corros. | ℈j |
| Spts. Vini, Rectif. | ℥viij. |

"Dissolve the guiac. and balsam in one-half of the spirit, and the corrosive sublimate in the other. Let the guiac. and balsam digest for several days; then pour off the clear liquor, mix with the sublimate, and add the oil. Dose, ten or twenty drops night and morning in a wineglass of water."

This combination was originally used by a Dr. Falk, of London, from whose book it was taken, some forty or fifty years ago, by the reporter's father. It was called by Dr. Falk tinct. antacrida.

Dr. Fenner directs the patient to begin a day or two before the expected period, and take twenty-five drops in an infusion of sage or sweetened water, night and morning, until the discharge is freely established; then cease till the next period. Of course, in very severe and obstinate cases, the remedy must be applied earlier, and the doses more frequently repeated. In this the practitioner is expected to exercise a judgment.—(*New Orleans Medical News and Hospital Gazette*, July, 1858, p. 300.)

*Dysmenorrhœa cured by Stramonium.* By A. P. MERRILL, M.D., Memphis, Tennessee.—This is described as a very obstinate case, which had resisted a variety of treatment.

The patient was tocated with the extract of stramonium, commenced in grain-doses, every three hours, ten days before the menstrual period. After five or six months of such treatment, the doses being reduced to half a grain, she became pregnant, and in excellent health.—(*New Orleans Medical News and Hospital Gazette*, October, 1858, p. 517.)

*Remedy for Dysmenorrhœa.* By W. O. BARKER, Omega, Texas.—This recipe appears in consequence of the author's having seen Dr. Fenner's cure for dysmenorrhœa published in *The New Orleans Medical News and Hospital Gazette.*

Dr. Barker tells us that, strongly impressed by the suspicion that dysmenorrhœa is a rheumatic affection, he has been in the habit of treating such cases successfully with the following preparation:—

| | |
|---|---|
| ℞.—G. Guiaci. | ℥j |
| Potassæ Nitrat. | ʒj |
| Flor. Sulphuris, | ʒj.—M. |

All to be well ground in a mortar, and put into one pint of brandy or good whisky, and after standing a few days, to be taken in tablespoonful doses morning and night, commencing one week before the menstrual period.

In retention or suppression of the catamenia, when the subject is otherwise healthy, the author recommends the following recipe:—

| | |
|---|---|
| ℞.—Ol. Terebinth. | f℥j |
| Ol. Sassafras. | ℥ss |
| Spt. Vini, Rectif. | f℥viij.—M. |

To be given in some simple tea, in doses of fʒj, *pro re nata.*—(*New Orleans Medical News and Hospital Gazette*, November, 1858, p. 595.)

*Removal of an Ovarian Tumor, in which the Ecraseur was used.* By JOHN L. ATLEE, M.D., Lancaster, Pennsylvania.—This operation is fully described. Dr. Atlee ascribes the rapid recovery of the patient, with much less constitutional disturbance than usually follows capital operations, to the use of the

écraseur, and the absence of any ligature involving the peritoneum.—(*The North American Medico-Chirurgical Review*, July, 1858, p. 698.)

*A Case of Ovariotomy successfully performed.* By ROBERT NELSON, M.D., New York.—This operation is likewise graphically described; but instead of the écraseur, the operator made use of strong hempen ligatures.

This lady, like the one operated upon by Dr. Atlee, made a complete and rapid recovery. The tumor weighed twenty-one pounds, and the abdominal incision for its removal measured twenty-two inches.—(*American Medical Monthly*, July, 1858, p. 14.)

*Two Cases of Ovarian Dropsy successfully treated.* By J. H. BREWER, M.D., Lawrenceburg, Indiana.—These two cases were treated by tapping and compression, as recommended and practiced by Mr. Brown, Surgeon-Accoucheur, of London. In addition to tapping and compression, Dr. Brewer informs us that he made use of iodine frictions, and the internal use of iodide of potassium, which he thinks materially aided the cure.—(*The Chicago Medical Journal*, May, 1858, p. 212.)

